# Assessing the carbonisation temperatures recorded by ancient charcoals for δ^13^C-based palaeoclimate reconstruction

**DOI:** 10.1038/s41598-022-17836-2

**Published:** 2022-08-29

**Authors:** C. Mouraux, F. Delarue, J. Bardin, T. T. Nguyen Tu, L. Bellot-Gurlet, C. Paris, S. Coubray, A. Dufraisse

**Affiliations:** 1 CNRS, EPHE, PSL, UMR 7619 METIS, Sorbonne Université, 4 place Jussieu, 75005 Paris Cedex 05, France; 2 CNRS, MNHN, UMR 7207 CR2P, Sorbonne Université, 4 place Jussieu, 75005 Paris, France; 3 CNRS, UMR 8233 MONARIS, Sorbonne Université, 4 place Jussieu, 75005 Paris, France; 4 UMR 7209 – AASPE-CNRS/MNHN, Archéozoologie, Archéobotanique: Sociétés, Pratiques et Environnements, CP56, 55 rue Buffon, 75005 Paris, France

**Keywords:** Biogeochemistry, Climate sciences

## Abstract

Ancient charcoal fragments, produced by the use of wood as fuel in archaeological contexts or during natural or anthropic forest fires, persist in soil and sediments over centuries to millennia. They thus offer a unique window to reconstruct past climate, especially palaeo-precipitation regimes thanks to their stable carbon isotope composition. However, the initial δ^13^C of wood is slightly modified as a function of the carbonisation temperature. Carbonisation-induced ^13^C fractionation is classically investigated through a transfer function between experimental carbonisation temperatures and the carbon content. This approach assumes that the carbon content is conservative through time in ancient charcoals and neglects the potential impact of post-depositional oxidation occurring in soils and sediments. In the present study, we first show that post-depositional oxidation can lead to a large underestimation of past carbonisation temperatures, thereby minimising the estimation of carbonisation-induced ^13^C fractionations and possibly biasing δ^13^C-based climate reconstructions. Secondly, by combining carbon content, Fourier-transform infrared and Raman spectroscopy, we propose a new framework to assess the carbonisation temperatures registered in ancient charcoals. This new framework paves the way to reassessing δ^13^C-based climate reconstruction.

## Introduction

The stable carbon isotope composition (δ^13^C) of woods is a function of the ^13^C/^12^C ratio of the initial wood, which depends on environmental conditions such as hydric stress, temperature, the isotope composition of CO_2_ and of the photosynthetic carbon fixation pathway^1–7^. The δ^13^C of woods (δ^13^C_wood_) is therefore widely used to infer past environmental changes^8–11^.

In contrast to wood remnants, charred woods, e.g. charcoals, persist in soils and sediments over centuries and millennia owing to their chemical structure dominated by aromatic units, which limits their biological and abiotic degradation with time^12^. Produced by the use of wood as fuel in archaeological contexts or during natural or anthropic forest fires, charcoals are commonly found in soils and sediments. The δ^13^C values of charcoals (δ^13^C_char_) have therefore been proposed as a useful proxy to assess changes in past environmental conditions—especially paleo-precipitation regimes—provided carbonisation-induced ^13^C fractionation is non-significant or is corrected^13–15^.

Expressed as Δ^13^C (Δ^13^C = δ^13^C_char_–δ^13^C_wood_), this ^13^C fractionation depends on the carbonisation temperatures undergone by the woods. ^13^C fractionation can be explained by the kinetic isotope effect and source-induced isotopic fractionations. In the specific case of wood carbonisation, it has been demonstrated that ^13^C fractionation was mainly driven by the thermal degradation of isotopically distinct biomolecules presenting distinct thermostabilities^16–18^. Several investigations have demonstrated that Δ^13^C modifications are directly related to the ^13^C isotope composition of thermolabile organic moieties degraded in the course of carbonisation^16–18^. Between ca. 180 °C and ca. 450 °C, the thermal degradation of ^13^C-enriched hemicelluloses and cellulose and the subsequent relative enrichment in ^13^C-depleted lignin have been shown to yield a reduction in bulk δ^13^C values close to the value determined on lignin^13,16–18^. In oak and pine woods, taxa commonly identified in European archaeological sites, carbonisation can therefore yield Δ^13^C reaching − 1.4 (600 °C) and − 2.0 ‰ (800 °C), respectively^17^. These Δ^13^C values are in the range of the δ^13^C variations determined in uncharred wood used to reconstruct past climate conditions^19,20^. Hence, Δ^13^C should be evaluated before using the δ^13^C of charcoals as a proxy to assess paleoclimatic changes.

Depending on the carbonisation temperature recorded by charcoals, Δ^13^C corrections are usually performed following a transfer function expressing Δ^13^C values as a function of the carbon content (%C) determined in experimentally produced charcoals^13,14,21^. Since carbonisation implies a loss of aliphatic units, oxygenated groups and other chemical groups containing heteroelements^22,23^, the chemical structure of the wood becomes progressively enriched in condensed aromatic units implying in turn, a well-known rise in %C with carbonisation temperature. However, using %C to assess carbonisation temperatures in ancient charcoals presupposes that %C is conservative through time. Expressed in mass percentage, %C does not only depend on the carbonisation temperatures undergone by woods but also on the modifications in the concentration of other organic and inorganic elements that can occur during post-depositional processes and related charcoal aging processes^24,25^. Additionally, no significant modification in δ^13^C_char_ values determined on charred woods was recorded after short-term burial experiments, suggesting that microbial and abiotic degradation has a negligible effect^26–28^. Hence, δ^13^C_char_ variations may strictly depend on carbonisation in contrast to %C.

Within soils and sediments, it is well known that the initial physicochemical properties (surface morphology, elemental composition, aromaticity, specific surface area, ion exchange capacity) are modified as a consequence of seasonal climate events (freeze–thaw and wetting–drying cycles), photochemical degradation, biodegradation and oxidation^29,30^. The oxidation of charcoals involves the formation of carboxylic and phenolic groups which in turn, implies a rise in %O compared to %C^24,29,31,32^. Hence, a subtle rise in the O content can induce a lowering of the %C. As cascade consequences, post-depositional oxidation may therefore underestimate carbonisation temperatures and Δ^13^C values, biasing in turn paleoclimatic reconstruction.

The Raman “thermometer” seems a promising tool to assess the carbonisation temperatures undergone by charred woods^33,34^. Raman spectroscopy has been demonstrated to be efficient in determining carbonisation temperatures in the 500–1000 °C range^33,34^. However, this Raman “thermometer” cannot estimate carbonisation temperatures between 350 and 450 °C, a range that has often been estimated in many ancient charcoals^13,14,21^. It has indeed been claimed that “*the usual range of carbonization temperatures appears to be relatively restricted in the fossil record"*^13^ (around 350–450 °C). This assertion seems, however, at odds with the 600–1000 °C temperature range undergone by wood in open fire^23,24^. This discrepancy suggests that a new methodological framework—independent of post-depositional processes—is necessary to (i) assess the carbonisation temperatures to which ancient charcoals were subjected and (ii) further optimise the use of δ^13^C_char_ as a palaeoenvironmental proxy.

The aims of this study were therefore (i) to illustrate the effect of oxidation on %C through a literature survey and (ii) to propose a framework to evaluate the carbonisation temperatures undergone by charcoals and Δ^13^C modifications. To this end, we investigated the %C, the ^13^C isotope composition and the chemical structure of charred *Quercus petraea* and *Pinus sylvestris* woods produced experimentally. The chemical structure of the charcoals was assessed by Fourier Transform infrared (FTIR) and Raman spectroscopies.

## Results and discussion

### Illustrating the potential effect of post-depositional oxidation on the determination of carbonisation temperatures by %C

**Figure 1:**
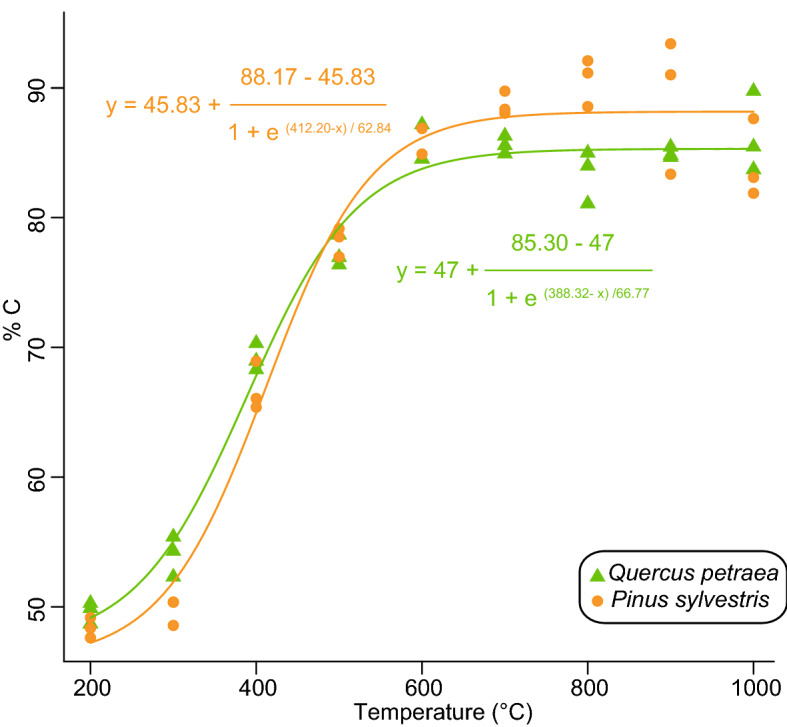
Relationships between carbonisation temperatures and %C in oak and pine charred woods.

Determined in fresh charcoals from *Q. petraea* and *P. sylvestris*, %C varies from 49.6 ± 0.8 (standard deviation) to 86.3 ± 3.1% and from 48.4 ± 0.8 to 90.6 ± 1.8%, respectively. %C is tightly related to pyrolysis temperatures, especially between 300 and 600 °C in accordance with a previous investigation^13^ (Fig. 1). Beyond 600 °C, %C remains roughly stable and does not allow any distinction between pyrolysis temperatures. The %C-based thermometer is therefore not suited to assess the carbonisation temperatures ranging between 600 and 1000 °C often measured in archaeological contexts^23,24^.

In addition to this first drawback, the use of %C to assess carbonisation temperatures may be biased by oxidation and the related increase in %O^29,30^. This was confirmed by a compilation of data from the literature (Fig. S1) evidencing the expected negative relationship between %C and %O in fresh charcoals resulting from the simultaneous rise in aromaticity and carbonisation temperature. As for fresh charcoals, aged charcoals also tend to present a negative relationship between %C and %O (Fig. S1). However, In contrast to fresh charcoals, aged charcoals are often characterised by higher and lower contributions of O and C, respectively, as a consequence of oxidation (Fig. S1). By comparing charcoals from an active and from pre‐industrial kilns situated in Belgium, Hardy et al.^35^ suggested that a 200-year post-depositional history can yield (i) an increase in %O ranging between 10.3 and 13% and (ii) a decrease in %C ranging between 27 and 29%. Comparison between fresh and Terra Preta charcoals suggests that oxidation can also strongly modify the O/C atomic ratio from ca. 0.1 to 0.6^29^. For example, applying such an oxidation to a fresh charcoal formed at 700 °C for which a %C and %O of ca. 85% and 10%, respectively should be determined (Fig. 1; Fig. S1), a theoretical %O of ca. 0.4% and a maximum %C of 56% were determined. We use the term “maximum” as, in the proposed calculation, the masses of C (85%) and of other elements (5%), except O, are considered unmodified. Following the relationship between %C and carbonisation temperatures (Fig. 1), this extreme oxidation would imply a carbonisation temperature of ca. 415 ± 32 °C although fresh charcoal was formed at 700 °C. These examples illustrate how the use of %C as a “paleothermometer” for ancient charcoals can substantially underestimate the carbonisation temperatures as a consequence of charcoal oxidation through time. However, it does not mean that oxidation systematically induces this bias but rather that additional tools are required to reconstruct past carbonisation temperatures. In the following, we will therefore discuss the contribution of FTIR and Raman spectroscopy to evaluate carbonisation temperatures in ancient charcoals.

### FTIR spectroscopy as a tool to evaluate low carbonisation temperatures

**Figure 2:**
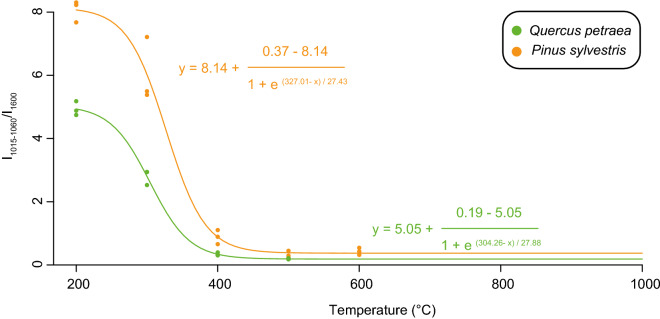
Relationships between the I_1015–1060_/I_1600_ and carbonisation temperatures in oak and pine charred woods.

In both charred woods from *Q. petraea* and *P. sylvestris*, an increasing pyrolysis temperature implies a reduction in all FTIR peak intensities (e.g. –OH, C = O, C–O–C, C-O, CH_X_) compared to the 1600 cm^−1^ band assigned to C=C in aromatics (Table S1; Fig. S2). As expected, most changes in the chemical structure of charred wood occurred between 300 and 400 °C (Fig. S2). Above 400 °C, FTIR spectra were dominated by the C=C intensity peak and no further structural modifications were recorded, suggesting that most of the thermolabile compounds—including cellulose and hemicelluloses—were thermally degraded (Fig. S2). These results are in line with previous investigations evidencing that pyrolysis between 300 and 500 °C entails the thermal decomposition of cellulose and hemicelluloses^36,37^. Among the observed FTIR peaks, those in the 3600–3000 cm^−1^ and in the 1015–1060 cm^−1^ regions can be of interest as they are usually assigned to O–H stretching and to a combination of C–O stretching and O–H deformation related to cellulose and hemicellulose, respectively (Table S1). As the FTIR absorption band in the 3600–3000 cm^−1^ region can be affected by O–H from water^38^, the thermal degradation of cellulose and hemicelluloses with carbonisation was therefore tracked here using the I_1015-1060_/I_1600_ ratio. Between 300 and 400 °C, the I_1015-1060_/I_1600_ ratio dramatically decreased from 4.9 ± 0.2 to 0.2 ± 0.02 and from 8.1 ± 0.3 to 0.3 ± 0.1 in *Q. petraea* and *P. sylvestris,* respectively, as a consequence of cellulose and hemicellulose thermal degradation (Fig. 2). Following this, a relationship between carbonisation temperatures and the I_1015–1060_/I_1600_ was observed (Fig. 2):

1 $$ {\text{I}}_{{1015 - 1060}} /{\text{I}}_{{{1600}}} { } = 5.05 + \frac{0.19 - 5.05}{{1 + e^{{\left( {304.26 - T^\circ C} \right)/27.88}} }}\;{\text{for}}\,{\text{charred}}\,{\text{oak}} $$

2 $$ {\text{I}}_{{1015 - 1060}} /{\text{I}}_{{{1600}}} { } = 8.14 + \frac{0.37 - 8.14}{{1 + e^{{\left( {327.01 - T^\circ C} \right)/27.43}} }}\;{\text{for}}\,{\text{charred}}\,{\text{pine}} $$

Most of the fossil charcoal record is thought to have been produced at low carbonisation temperatures ranging between 350 °C and 450°C^13,14,21^. If these estimated carbonisation temperatures are correct, a significant content of cellulose and hemicellulose should still be preserved in these ancient charcoals provided that the remnants of cellulose and hemicellulose were not degraded during post-depositional processes. As charcoals are increasingly enriched in condensed aromatic rings during carbonisation, they have long been thought to be insensitive or weakly sensitive to biodegradation because of their carbon structure and possibly, the presence of carbonisation by-products that inhibit enzymatic activities^39^. However, there is compelling evidence for a significant biodegradation of charcoal with time and therefore, a modification of its initial geochemical signatures^40,41^. As charcoals are often considered as being composed of a highly condensed carbon structure, most investigations into biodegradation have focused on the oxidation of the aromatic structure. However, our results and previous investigations demonstrated that significant amounts of polysaccharides can be preserved within charcoals especially those formed at low carbonisation temperature^42–44^. This suggests that a readily available substrate may be still available for microbial activities. To date, the biodegradation of remnants of hemicellulose/cellulose within charcoal remains undocumented. Considering this gap in extant knowledge but also the overlapping of FTIR bands due to the occurrence of the inorganic fraction^45^, we suggest that the application of the FTIR thermometer should be restricted to fresh charcoals devoid of ash. Nonetheless, the occurrence of FTIR bands typically related to the preservation of cellulose/hemicellulose is an essential observation for diagnosing a carbonisation temperature below 400 °C in ancient charcoals. In other words, and after a careful rinsing to avoid carbohydrates originating from biofilms, such FTIR bands should be observed in most of the fossil charcoal record which was proposed to have been produced at ca. 400 °C—if preserved against soil/sediment microbial decomposition activities^13,14,21^.

### Raman thermometry

**Figure 3:**
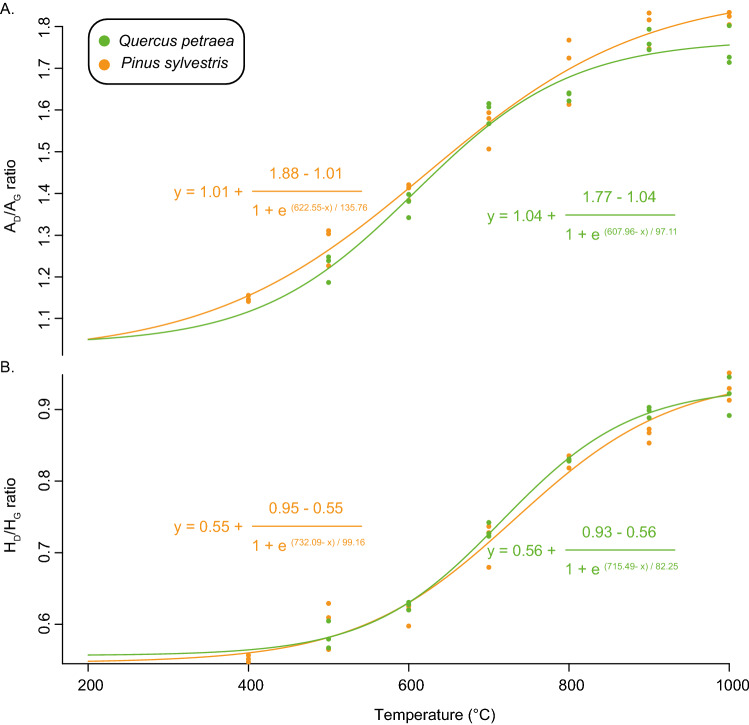
Relationships between (**A**) the A_D_/A_G_ and (**B**) H_D_/H_G_ ratios and carbonisation temperatures in oak and pine charred woods.

In contrast to archaeological charcoals putatively produced at low carbonisation temperatures, several investigations suggested that some archaeological charcoals underwent a carbonisation temperature ranging between 600 and 1000 °C (house fires, pottery kilns, etc.). In this temperature range, Raman spectroscopy has been demonstrated to be efficient in determining the carbonisation temperature^23,33,34^. These pioneering works suggested that Raman-derived parameters can assess the carbonisation temperature—ranging between 500 and 1200 °C—undergone by both charred pine and oak woods. In this study, the fluorescence level was too high to record any Raman spectra in *Q. petraea* and in *P. sylvestris*, respectively, below 400 °C and 500 °C. This may be a consequence of the aliphatic content remaining in charred wood at such carbonisation temperatures as suggested by FTIR spectra (see the occurrence of CH_2_ bonds in the 2850–2920 cm^−1^ FTIR band region; Fig. S2). At and beyond these carbonisation temperatures, we observed the well-known “carbonisation trend” determined in a wide range of carbonaceous materials subjected to thermal degradation^23,46,47^ (Fig. S3). This carbonisation trend consists in a simultaneous rise (i) in the A_D_/A_G_ ratio from 1.15 to 1.82 and from 1.22 to 1.77 and (ii) in the H_D_/H_G_ ratio from 0.55 to 0.93 and from 0.58 to 0.93 in both *Q. petraea* and in *P. sylvestris* charred woods, respectively (Fig. 3).

A_D_/A_G_ and H_D_/H_G_ ratios can both be used to assess carbonisation temperatures between 400 and 1000 °C in the studied charred woods.

3 $$ {\text{AD}}/{\text{AG }} = 1.04 + \frac{1.77 - 1.04}{{1 + e^{{\left( {607.96 - T^\circ {\text{C}}} \right)/97.11}} }}\;{\text{for}}\,{\text{charred}}\,{\text{oak}} $$

4 $$ {\text{AD}}/{\text{AG}} = 1.01 + \frac{1.88 - 1.01}{{1 + e^{{\left( {622.55 - T^\circ {\text{C}}} \right)/135.76}} }}\;{\text{for}}\,{\text{charred}}\,{\text{pine}} $$

5 $$ {\text{HD}}/{\text{HG}} = 0.56 + \frac{0.93 - 0.56}{{1 + e^{{\left( {715.49 - T^\circ {\text{C}}} \right)/82.25}} }}\;{\text{for}}\,{\text{charred}}\,{\text{oak}} $$

6 $$ {\text{HD}}/{\text{HG}} = 0.55 + \frac{0.95 - 0.55}{{1 + e^{{\left( {732.09 - T^\circ {\text{C}}} \right)/99.16}} }}\;{\text{for}}\,{\text{charred}}\,{\text{pine}} $$

Based on the integration of the complex and broad D band, the A_D_/A_G_ ratio is highly sensitive to the modification in the shape of D sub-bands. Occurring at ca. 1350 cm^−1^, the D1-band is related to heteroatoms, vacancies and structural defects^23,48^ (Fig. S3). During carbonisation, the D1 band intensity therefore irreversibly increases as a consequence of the resulting increase in the size of the polyaromatic layers^34^. Moreover, the D5 sub-band, which occurs at ca. 1450 cm^−1^ (Fig. S3), is thought to diagnose the presence of aliphatic hydrocarbons (i) entrapped within the nanoporosity of the macromolecular network and (ii) degraded in the course of carbonisation^23,49^.

### Reconsidering the determination of past carbonisation temperatures to correct δ^13^C in ancient charcoals.

**Figure 4:**
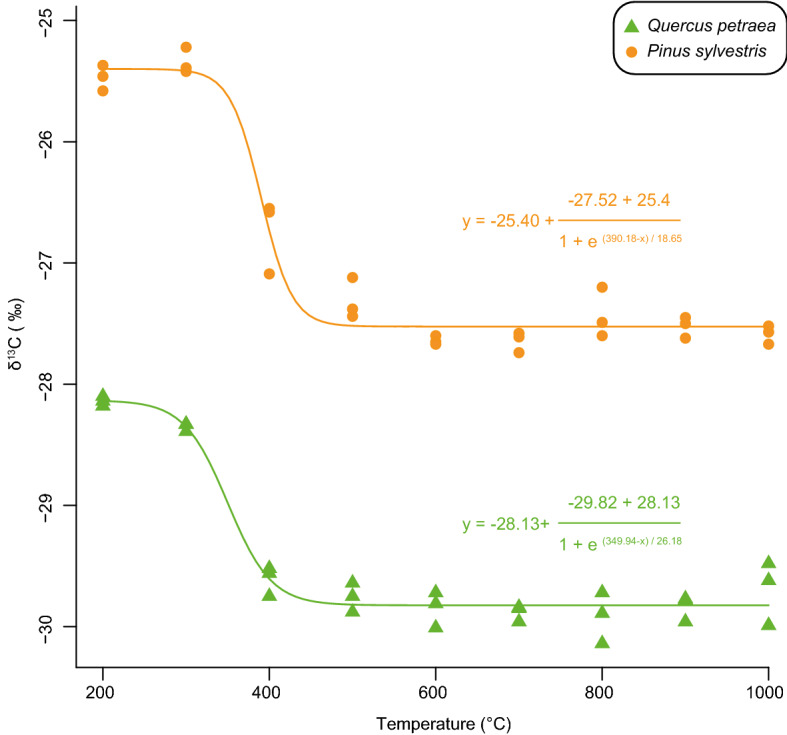
Modifications of δ^13^C_char_ values with carbonisation temperatures in charred oak and pine charred woods.

A decrease in δ^13^C_char_ values during charring is a well-known process^16–18^. Most of the carbonisation-induced δ^13^C modifications take place below a temperature of 400 °C (Fig. 4), a temperature at which most of the cellulose and hemicellulose—relatively enriched in ^13^C—is thermally degraded^13,16–18^. From 200 to 600 °C, δ^13^C_char_ values decrease from − 28.1 ± 0.04 to -29.8 ± 0.1‰ and from − 25.5 ± 0.1 to − 27.6 ± 0.04 ‰ in charred oak and pine woods, respectively (Fig. 4). Between 600 and 1000 °C, no clear modifications of the δ^13^C values were observable (Fig. 4). In this latter temperature range, δ^13^C_char_ exhibits values from − 29.9 ± 0.2 to − 29.7 ± 0.3 ‰ and from − 27.4 ± 0.2 to − 27.6 ± 0.1 ‰ in charred oak and pine woods, respectively (Fig. 4). Our results suggest that a maximum Δ^13^C value of 1.8 and 2.2‰ is reached for charred oak and pine woods, respectively (Fig. 4).

Classically, the effect of carbonisation is corrected following a transfer function using %C to assess carbonisation temperatures^13,14,21^. However, as stressed before, %C can be potentially biased by the oxidation occurring during post-depositional processes but also be limiting to assess carbonisation temperatures above 600 °C. Since the application of the FTIR thermometer is restricted to fresh charcoal, only Raman-derived parameters seem appropriate to determine carbonisation temperatures (as the studied Raman parameters seem to be insignificantly modified by post-depositional processes^50^). However, as the typical D and G bands arise after 400 °C—the temperature at which most of the carbonisation-induced ^13^C fractionation occurs—Raman thermometers cannot be used to propose a transfer function between δ^13^C_char_ and carbonisation temperature or δ^13^C_wood_. Nonetheless, the combination of %C, FTIR and Raman spectroscopies can provide a framework to assess and/or reassess the carbonisation temperatures undergone by charred oak and pine woods following the scheme provided in Fig. S4.

FTIR spectroscopy is the first step in this framework. After a thorough preliminary treatment to avoid the occurrence of exogenous OM, the observation of the FTIR band at ca. 1045 cm^−1^—related to C-O bonds in hemicellulose and cellulose—shows that carbonisation temperatures were below 400 °C. Nonetheless, one should keep in mind that a subtle modification in the %O can lead to a large underestimation of carbonisation temperatures in particular in the 300–400 °C range where %C and δ ^13^C are strongly modified. In addition, it is worth noting that carbonisation below 400 °C cannot be ruled out in the absence of both hemicellulose/cellulose FTIR bands because of microbial decomposition occurring during post-depositional processes. In this respect, the absence of Raman spectra (below 400 and 500 °C for oak and pine charred woods) is a key observation and should have profound implications for reassessing the carbonisation temperatures of ancient charcoals used for palaeoclimate reconstruction. If most charcoals in the fossil record were indeed produced below a temperature of ca. 400 °C, they should lack the D and G bands observed with Raman spectroscopy. If this condition is not fulfilled, it would suggest that Δ^13^C may be underestimated by up to 1‰, a value in the range of the climate-induced δ^13^C variations determined in uncharred wood^19,20^. Above 600 °C, ^13^C modifications reach a maximum value, implying a direct correction of carbonisation-induced ^13^C fractionation through a simple subtraction. This is the only case for which a univocal correction of δ^13^C values is possible (Fig. S4). Hence, in archaeological settings where charcoals were formed in a variety of contexts (on open fires, house burning, kilns, etc.) and registered carbonisation temperatures between 600 and 1000 °C, δ^13^C_char_ can be directly compared to assess palaeo-precipitation regimes provided they were measured on a single tree species. A simple subtraction of δ^13^C_char_ by the maximum Δ^13^C value can also be considered to assess δ^13^C_wood_ according to δ^13^C_char_.

In the case of charcoals that experienced carbonisation temperatures below 600 °C, such a scheme cannot be directly applied. We therefore propose that these charred woods should be recarbonised until reaching the carbon structural order observed here at a temperature of 600 °C. By taking advantage of the irreversibility of chemical changes occurring during carbonisation, this recarbonisation step implies that the maximum Δ^13^C value will be theoretically reached. In addition to allowing a direct comparison between δ^13^C_char_ determined in charcoals from a single species, the main advantage of this simplistic approach is that it (i) avoids all uncertainties related to oxidation and the use of the %C “thermometer” and (ii) minimises error propagation related to the determination of Δ^13^C values in the course of carbonisation.

## Conclusion

Determining the evolution of past climate is of fundamental interest to understand interactions between past societies and climate change. To this end, charcoal macro-remains, which are the most frequent modes of wood conservation in archaeological sites, thus represent a key record. Their ^13^C isotope composition can yield valuable information provided the effects of carbonisation and of post-depositional oxidation are constrained. Here we show that post-depositional oxidation—a subsequent rise in %O—can bias the determination of carbonisation temperatures through %C. By reducing the %C, post-depositional processes can therefore lead to an underestimation of carbonisation temperatures implying in turn, an inappropriate correction of carbonisation-induced ^13^C fractionation. By studying the chemical structure of *Q. petraea* and *P. sylvestris* charred woods formed between 200 and 1000 °C in an inert atmosphere, our results tend to question previous evidence for carbonisation temperatures below 400 °C in some archaeological charcoals. Two main criteria were identified to ensure that these archaeological charcoals were indeed formed below 400 °C:

- Preservation of cellulose and hemicellulose observed through infrared spectroscopy provided they were preserved against biodegradation;
- Lack of the typical Raman D and G bands classically observed in Raman spectroscopy.

Above an apparent carbonisation temperature of 600 °C, Δ^13^C remains stable, suggesting that no ^13^C corrections or a direct correction of carbonisation through the simple subtraction of δ^13^C_char_ by the maximum Δ^13^C is required. Following our conclusions, we suggest that a thorough examination of the chemical structure of ancient charcoals is required to assess and reassess past climate changes using the ^13^C isotope composition.

## Methods

### Experimental carbonisation

Charcoal samples were produced experimentally in the laboratory from crushed oak (*Q. petraea*) and pine (*P. sylvestris*) woods. Powdered wood was chosen to limit any physical roughness and chemical heterogeneity that might induce a change in the mean response to carbonisation. *Q*. *petraea* and *P. sylvestris* were subjected to pyrolysis in a furnace pyrolyser under N_2_ flow (O_2_-free atmosphere). The samples (ca. 200 mg) were placed in a quartz tube plugged with quartz wool that was then heated for 1 h at 200, 300, 400, 500, 600, 700, 800, 900 and 1000 °C. It is worth mentioning that heating durations varied between 20 min to 15 h in previous carbonisation experiments^16,17,51,52^. A previous investigation demonstrated that a heating duration above 1 h has little effect on the degree of aromaticity of charcoals recorded with Raman spectroscopy^33^. In contrast, there is still no clear evidence that heating durations below 1 h are enough to reach a similar degree of aromaticity. Hence, a heat duration of 1 h was chosen to minimise the effect of heat duration on the selected carbonisation temperatures. All pyrolysis were performed in triplicate.

### Carbon concentration and δ^13^C values

Approximately 0.2–0.3 mg of charcoal were combusted using an elemental analyser (Thermo Fisher Scientific Flash, 2000) coupled to an isotope ratio mass spectrometer (Thermo Fisher Scientific Delta V advantage) to determine both %C and δ^13^C. Uncharred oak wood, tyrosine and urea were used as internal standards (standard deviation of ca. 0.1‰). Internal standard true values, measured values and standard deviations are provided in supplementary Table 2.

### Fourier transform infrared spectroscopy

FTIR spectroscopy was performed by attenuated total reflectance FTIR spectroscopy using a Bruker Tensor 27 spectrometer. The powdered uncharred and charred materials were placed directly on a germanium crystal. FTIR spectra were acquired by 64 scans at a 2 cm^−1^ resolution over the range 4000–600 cm^−1^. All spectra were corrected for water vapour, CO_2_ and for differences in depth of beam penetration at different wavelengths (ATR correction; Opus software). All spectra were then normalised. For each spectrum, standardisation involved a subtraction of the minimum absorption value applied to the whole spectrum followed by a multiplication—applied on the whole spectrum—to obtain a similar spectral maximum absorbance value for all uncharred and charred woods^53^. The I_1015-1060_/I_1600_ intensity ratio was then computed to track the fate of cellulose/hemicelluloses during carbonisation at low pyrolysis temperature. Assignments of FTIR absorption bands are summarised in supplementary Table 1.

### Raman spectroscopy

Raman spectroscopy was performed using a MR800 Horiba Jobin Yvon spectrometer equipped with a 514.5 nm green laser. The laser was focused on the sample with a 50 × objective. The spectrometer was first calibrated with a silicon standard before the analytical session (matching at 520.5 cm^−1^). A laser power below 1 mW was used to prevent any thermal alteration during spectrum acquisition^54,55^. Spectrum acquisition was achieved after three iterations using a time exposure of 180 s (spectral resolution of 2 cm^−1^). For each sample, we determined the Raman shift intensity in the 600 to 2300 cm^−1^ spectral window including the first-order disorder (D) and graphite (G) peaks centred at about 1350 and 1600 cm^−1^, respectively. The D band is a complex band assigned to defects in the aromatic structure (e.g. functional groups, heteroatoms and vacancies) and to amorphous carbon tightly associated with the macromolecular network^48,56^. The G band is ascribed to in-plane stretchings of C=C bonds within graphene-like clusters^48^. After linear baseline correction, two Raman-derived parameters were computed: the A_D_/A_G_ and the H_D_/H_G_ ratios. The A_D_/A_G_ ratio is the ratio between the integrated D (between 1000 and 1500 cm^−1^) and G areas (between 1500 and 1800 cm^−1^).The H_D_/H_G_ ratio corresponds to the ratio between the maximum peak intensity of the D and G peaks.

### Statistics

To evaluate the relationship between carbonisation temperatures and the chemical structure of charcoals, regression models were used. All the variables under study (i.e. the %C, the ^13^C isotope composition, the I_1015-1060_/I_1600_ intensity ratio, the A_D_/A_G_ and the H_D_/H_G_ ratios) vary with the temperature following S-shaped curves. The generalised logistic function (also known as Richard’s function) was therefore used to perform regressions.

7 $$ Y\left( {T^\circ {\text{C}}} \right) = a + \frac{b - a}{{1 + e^{{\left( {xm - T^\circ {\text{C}}} \right)/s}} }} $$

This function depends on five parameters: *a*, the asymptote for low temperatures, is the initial condition before carbonisation; *b*, the asymptote for high temperatures, corresponds to the state of the sample for a theoretical infinite temperature; *xm*, corresponds to the middle of the S-curve, in other words, the temperature where half of the process has been completed; *s* quantifies the curvature of the S-curve. To determine the least-squares estimates of the parameters, we used the *nls* function of the *stats* package^57^. To estimate the error in predicting the responses given a temperature, 95% prediction intervals were computed using the *propagate* package. However, when one wants to use the regressions done herein to predict a temperature given a response (e.g. obtaining a temperature given an A_D_/A_G_ ratio), the calibration error has to be added. For that purpose, we used the delta method as described in Huet et al.^58^ which has the advantage of accounting for both the variability of the response and the uncertainty about the calibration curve.

Based on literature data, a regression between the oxygen content (%O) and %C determined in fresh and aged charcoals was also conducted (Fig. S1). The regression of the %O against %C is linear in the form y = ax + b and was done with the *lm* function.

## Supplementary Information

Supplementary Information.

## Data Availability

Data are provided in supplementary table 3.
