# Supplementary material for: Assessing the carbonisation temperatures recorded by ancient charcoals for δ13C-based palaeoclimate reconstruction
Source: Sci Rep. 2022 Aug 29;12:14662. doi: 10.1038/s41598-022-17836-2 (PMC9424292; doi:10.1038/s41598-022-17836-2)
Supplement: Supplementary file 1 — Supplementary Information. [file 41598_2022_17836_MOESM1_ESM.docx]

**Supplementary Figure S1**: Compilation of data from the literature presenting %C and %O determined in fresh and in (artificially and naturally) aged charcoals^1-15^. “Fresh charcoals” refers to charcoals produced experimentally that were not subjected to any post-depositional processes.


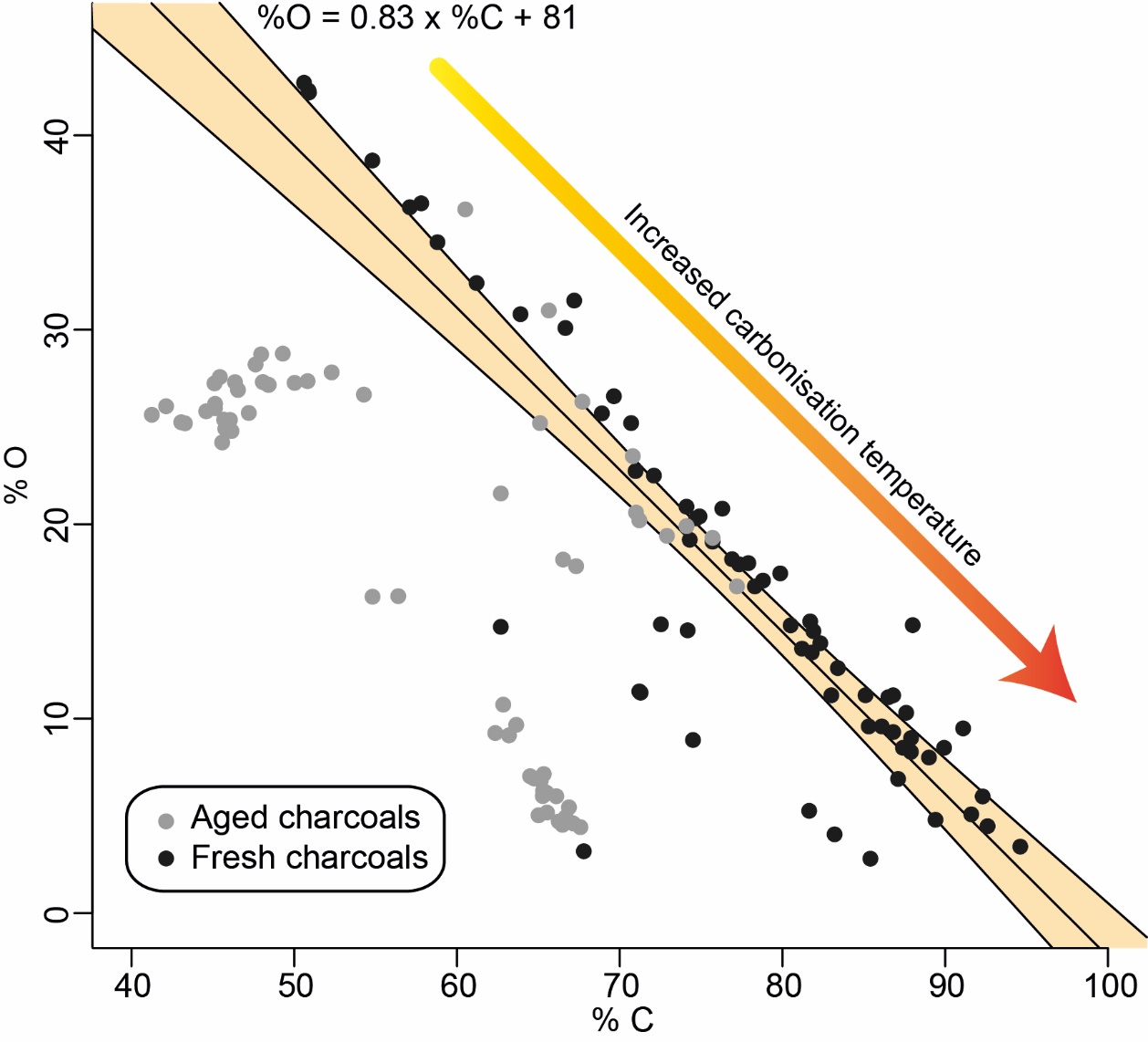


**Supplementary Figure S2:** FTIR spectra measured on uncharred and charred oak woods.


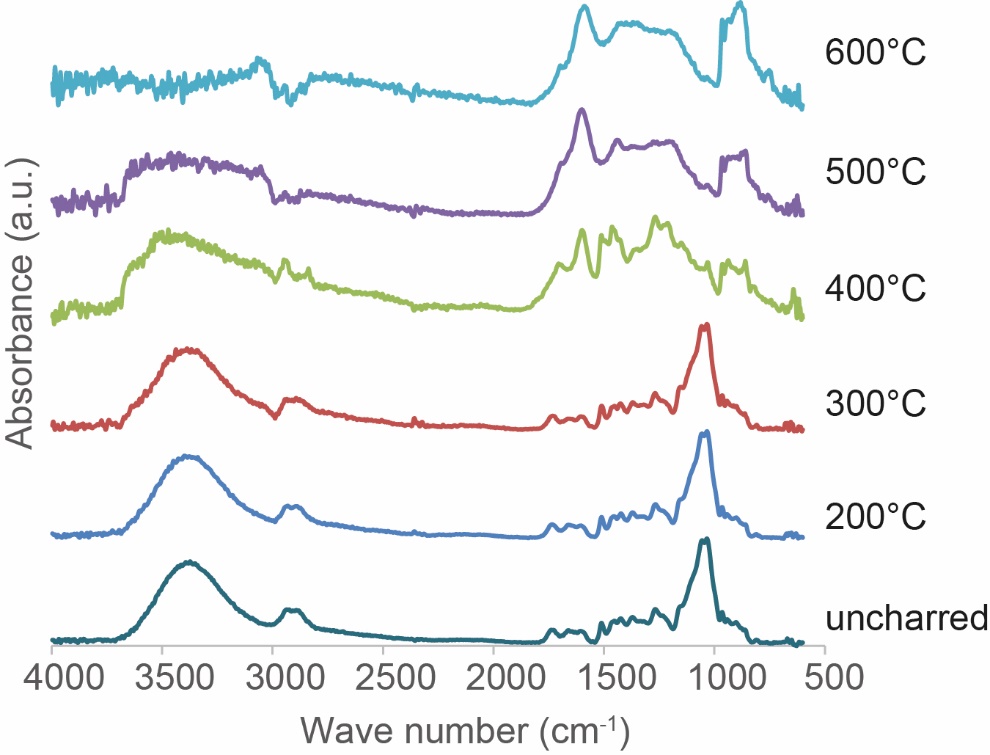


**Supplementary Figure S3:** Raman spectra measured on charred oak woods.


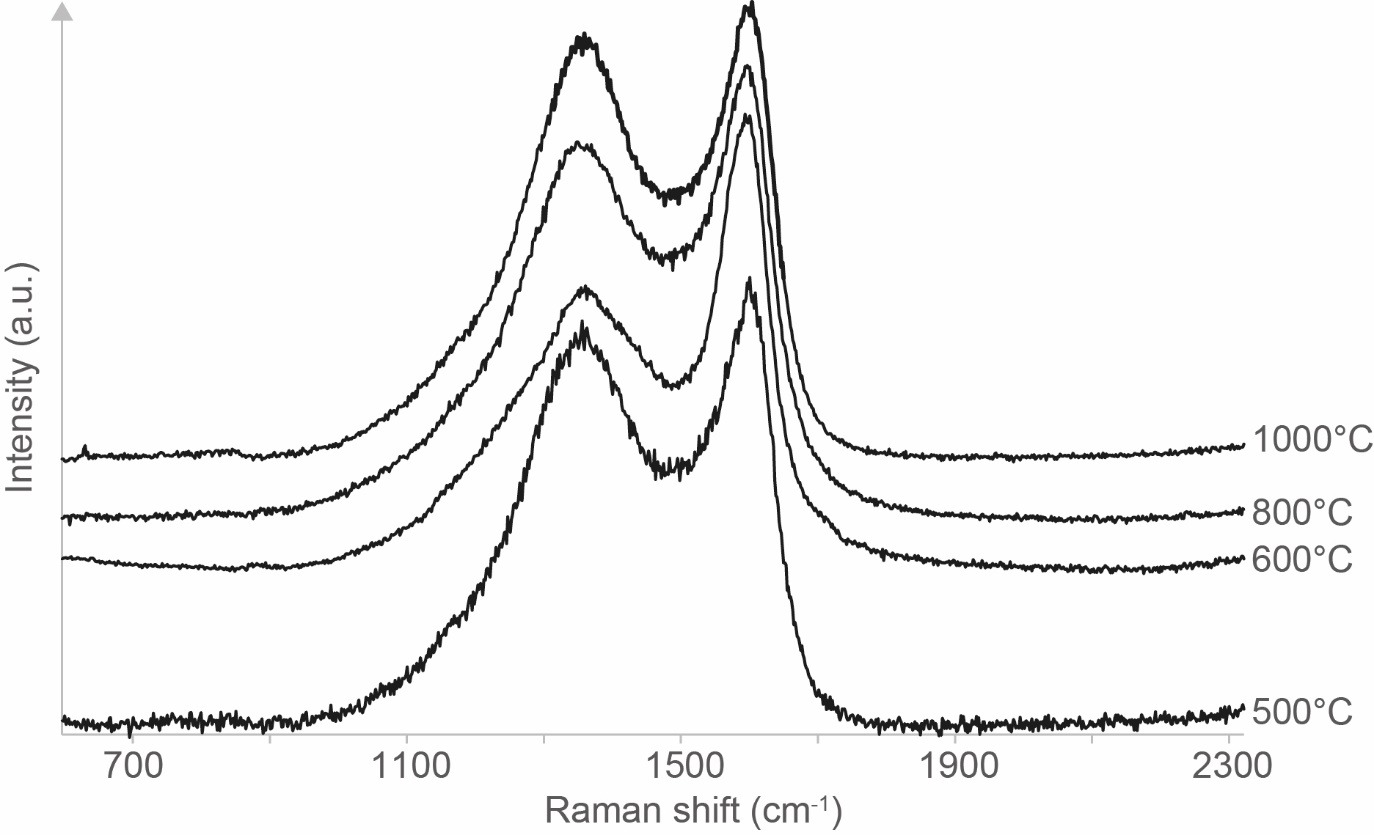


**Supplementary Figure S4:** General scheme to assess the carbonisation degree undergone by ancient charcoals using FTIR and Raman spectroscopy (C.T.: carbonisation temperature).


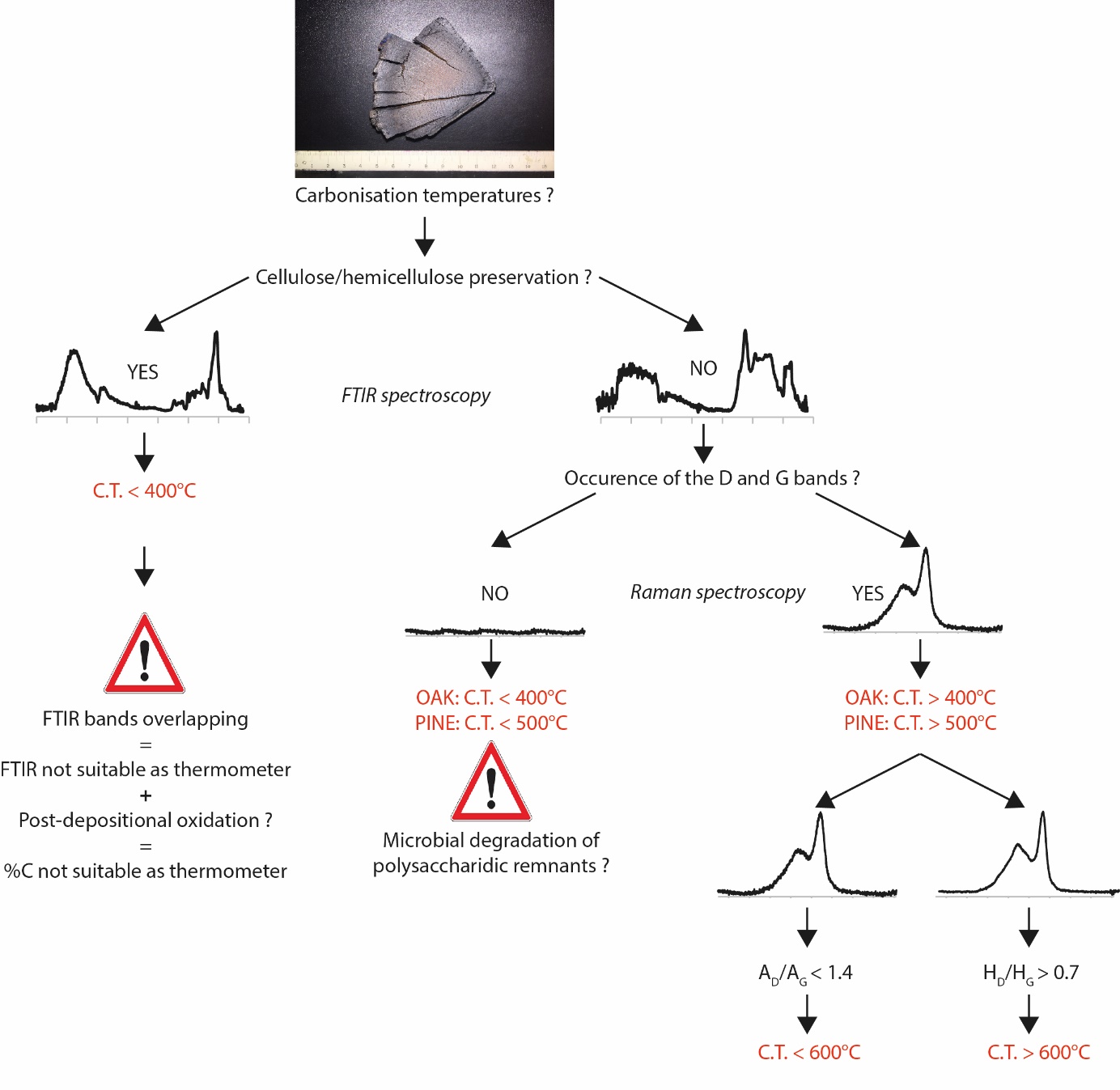


**Supplementary table 1**: Assignment of the main FTIR absorption bands^16-19^.

| **Wave number (cm^-1^)** | **Base group and vibration mode** |
| --- | --- |
| 3360-3430 | Intramolecular hydrogen bonds in cellulose |
| 2920 | Antisymmetric CH_2_ |
| 2850 | Symmetric CH_2_ |
| 1739 | C-O vibration of esters, ketones, aldehydes in hemicellulose |
| 1640 | C=O stretching conjugated to aromatic ring |
| 1600 | aromatic skeletal vibrations of lignin and C=O stretching |
| 1595 | aromatic skeletal vibrations and C=O stretching of lignin |
| 1510 | Aromatic skeletal stretching |
| 1505 | aromatic skeletal vibrations and C=O stretching of lignin |
| 1464 | CH_2_ asymmetric stretching, symmetric stretching, and in-plane scissoring vibrations |
| 1425 | Aromatic skeletal combined with C-H in-plane deforming and stretching |
| 1375 | Aliphatic C-H stretching in methyl and phenol OH |
| 1330 | vibration in C-H and stretching in C-O related to syringyl ring |
| 1317 | Condensation of guaiacyl and syringyl units, syringyl unit and CH_2_ bending stretching |
| 1269 | Guaiacyl ring vibrations and C-O stretching lignin |
| 1230 | C-C, C-O and C=O stretching |
| 1160 | C-O-C stretching in pyranose rings, C=O in aliphatic groups |
| 1111 | Antisymmetric in-phase ring stretching |
| 1015-1060 | C-O valence vibrations in cellulose |
| 1041 | C-O and C-C rings changes in xyloglucan |
| 1035 | C-O stretching mainly of primary alcohols in cellulose |
| 897 | in-plane symmetric vibration of C-H in cellulose |

**Supplementary table 2**: %C and δ^13^C (‰, V-PDB) measurements on standards

|  | **Tyrosine** | **Urea** | **Uncharred oak wood** |
| --- | --- | --- | --- |
| True %C value | 69.3 | 59,7 | 48.4 |
| Analysed %C value | 68.6 | 63.4 | 47.8 |
| Standard deviation | 0.7 | 0.9 | 0.5 |
| True δ^13^C value | -28.2 | -23.2 | -28.1 |
| Analysed δ^13^C value | -27.7 | -22.7 | -28.1 |
| Standard deviation | 0.1 | 0.1 | 0.1 |

**Supplementary table 3**: %C, δ^13^C (‰), Raman- and FTIR-derived parameters determined on oak (Q) and pine (P) woods charred between 200 and 1000°C. (n.d.: not determined)

|  |  |  |  |  |  |  |  |
| --- | --- | --- | --- | --- | --- | --- | --- |
| **Species** | **Replicate** | **Temperature** | **%C** | **δ^13^C** | **I_1015-1060_/I_1600_** | **A_D_/A_G_** | **H_D_/H_G_** |
| Q | 1 | 200 | 48.7 | -28.1 | 4.7 | n.d. | n.d. |
| Q | 2 | 200 | 49.9 | -28.2 | 4.9 | n.d. | n.d. |
| Q | 3 | 200 | 50.3 | -28.1 | 5.2 | n.d. | n.d. |
| Q | 1 | 300 | 55.4 | -28.3 | 2.5 | n.d. | n.d. |
| Q | 2 | 300 | 54.3 | -28.4 | 2.9 | n.d. | n.d. |
| Q | 3 | 300 | 52.3 | -28.3 | 2.9 | n.d. | n.d. |
| Q | 1 | 400 | 69.0 | -29.5 | 0.3 | 1.2 | 0.6 |
| Q | 2 | 400 | 70.3 | -29.8 | 0.4 | 1.1 | 0.6 |
| Q | 3 | 400 | 68.3 | -29.6 | 0.3 | 1.1 | 0.5 |
| Q | 1 | 500 | 76.4 | -29.6 | 0.2 | 1.3 | 0.6 |
| Q | 2 | 500 | 76.9 | -29.8 | 0.2 | 1.2 | 0.6 |
| Q | 3 | 500 | 78.7 | -29.9 | 0.2 | 1.3 | 0.6 |
| Q | 1 | 600 | 87.2 | -29.7 | n.d. | 1.4 | 0.6 |
| Q | 2 | 600 | 84.5 | -29.8 | n.d. | 1.4 | 0.6 |
| Q | 3 | 600 | 84.5 | -30.0 | n.d. | 1.4 | 0.6 |
| Q | 1 | 700 | 86.3 | -29.9 | n.d. | 1.5 | 0.7 |
| Q | 2 | 700 | 84.9 | -29.8 | n.d. | 1.6 | 0.7 |
| Q | 3 | 700 | 85.6 | -30.0 | n.d. | 1.6 | 0.7 |
| Q | 1 | 800 | 85.0 | -30.1 | n.d. | 1.8 | 0.8 |
| Q | 2 | 800 | 84.0 | -29.9 | n.d. | 1.7 | 0.8 |
| Q | 3 | 800 | 81.1 | -29.7 | n.d. | 1.6 | 0.8 |
| Q | 1 | 900 | 84.7 | -30.0 | n.d. | 1.7 | 0.9 |
| Q | 2 | 900 | 85.5 | -29.8 | n.d. | 1.8 | 0.9 |
| Q | 3 | 900 | 84.8 | -29.8 | n.d. | 1.8 | 0.9 |
| Q | 1 | 1000 | 83.7 | -30.0 | n.d. | 1.8 | 1.0 |
| Q | 2 | 1000 | 85.5 | -29.5 | n.d. | 1.8 | 0.9 |
| Q | 3 | 1000 | 89.8 | -29.6 | n.d. | 1.8 | 0.9 |
| P | 1 | 200 | 47.6 | -25.5 | 8.2 | n.d. | n.d. |
| P | 2 | 200 | 48.4 | -25.6 | 7.7 | n.d. | n.d. |
| P | 3 | 200 | 49.2 | -25.4 | 8.3 | n.d. | n.d. |
| P | 1 | 300 | 50.4 | -25.2 | 5.5 | n.d. | n.d. |
| P | 2 | 300 | 50.4 | -25.4 | 7.2 | n.d. | n.d. |
| P | 3 | 300 | 48.6 | -25.4 | 5.4 | n.d. | n.d. |
| P | 1 | 400 | 66.1 | -26.6 | 0.9 | n.d. | n.d. |
| P | 2 | 400 | 68.9 | -27.1 | 0.7 | n.d. | n.d. |
| P | 3 | 400 | 65.4 | -26.6 | 1.1 | n.d. | n.d. |
| P | 1 | 500 | 77.0 | -27.1 | 0.3 | 1.2 | 0.6 |
| P | 2 | 500 | 78.5 | -27.4 | 0.2 | 1.2 | 0.6 |
| P | 3 | 500 | 79.2 | -27.4 | 0.4 | 1.2 | 0.6 |
| P | 1 | 600 | 84.9 | -27.6 | 0.3 | 1.4 | 0.6 |
| P | 2 | 600 | 86.9 | -27.7 | 0.4 | 1.4 | 0.6 |
| P | 3 | 600 | 86.9 | -27.7 | 0.5 | 1.3 | 0.6 |
| P | 1 | 700 | 88.1 | -27.7 | n.d. | 1.6 | 0.7 |
| P | 2 | 700 | 88.4 | -27.6 | n.d. | 1.6 | 0.7 |
| P | 3 | 700 | 89.8 | -27.6 | n.d. | 1.6 | 0.7 |
| P | 1 | 800 | 88.6 | -27.5 | n.d. | 1.6 | 0.8 |
| P | 2 | 800 | 91.2 | -27.2 | n.d. | 1.6 | 0.8 |
| P | 3 | 800 | 92.1 | -27.6 | n.d. | 1.6 | 0.8 |
| P | 1 | 900 | 93.4 | -27.5 | n.d. | 1.8 | 0.9 |
| P | 2 | 900 | 83.4 | -27.5 | n.d. | 1.7 | 0.9 |
| P | 3 | 900 | 91.0 | -27.6 | n.d. | 1.8 | 0.9 |
| P | 1 | 1000 | 87.6 | -27.7 | n.d. | 1.7 | 0.9 |
| P | 2 | 1000 | 83.1 | -27.6 | n.d. | 1.8 | 0.9 |
| P | 3 | 1000 | 81.9 | -27.5 | n.d. | 1.8 | 0.9 |

**Supplementary references**:

1. Cheng, C.-H., Lehmann, J., Thies, J. E., Burton, S. D. & Engelhard, M. H. Oxidation of black carbon by biotic and abiotic processes. *Organic Geochemistry* **37**, 1477–1488 (2006).

2. Cordero, T., Marquez, F., Rodriguez-Mirasol, J. & Rodriguez, J. J. Predicting heating values of lignocellulosics and carbonaceous materials from proximate analysis. *Fuel* **80**, 1567–1571 (2001).

3. Crombie, K., Mašek, O., Sohi, S. P., Brownsort, P. & Cross, A. The effect of pyrolysis conditions on biochar stability as determined by three methods. *GCB Bioenergy* **5**, 122–131 (2013).

4. Enders, A., Hanley, K., Whitman, T., Joseph, S. & Lehmann, J. Characterization of biochars to evaluate recalcitrance and agronomic performance. *Bioresource Technology* **114**, 644–653 (2012).

5. Hardy, B. *et al.* Long term change in chemical properties of preindustrial charcoal particles aged in forest and agricultural temperate soil. *Organic Geochemistry* **107**, 33–45 (2017).

6. Huff, M. D., Kumar, S. & Lee, J. W. Comparative analysis of pinewood, peanut shell, and bamboo biomass derived biochars produced via hydrothermal conversion and pyrolysis. *Journal of Environmental Management* **146**, 303–308 (2014).

7. Keiluweit, M., Nico, P. S., Johnson, M. G. & Kleber, M. Dynamic Molecular Structure of Plant Biomass-Derived Black Carbon (Biochar). *Environ. Sci. Technol.* **44**, 1247–1253 (2010).

8. Mukome, F. N. D., Zhang, X., Silva, L. C. R., Six, J. & Parikh, S. J. Use of Chemical and Physical Characteristics To Investigate Trends in Biochar Feedstocks. *J. Agric. Food Chem.* **61**, 2196–2204 (2013).

9. Nelissen, V. *et al.* Short-Term Effect of Feedstock and Pyrolysis Temperature on Biochar Characteristics, Soil and Crop Response in Temperate Soils. *Agronomy* **4**, 52–73 (2014).

10. Quan, G. *et al.* Effects of laboratory biotic aging on the characteristics of biochar and its water-soluble organic products. *Journal of Hazardous Materials* **382**, 121071 (2020).

11. Ren, X., Sun, H., Wang, F., Zhang, P. & Zhu, H. Effect of aging in field soil on biochar’s properties and its sorption capacity. *Environmental Pollution* **242**, 1880–1886 (2018).

12. Spokas, K. A. Review of the stability of biochar in soils: predictability of O:C molar ratios. *Carbon Management* **1**, 289–303 (2010).

13. Spokas, K. A. Reicosky, D.C. Impacts of sixteen different biochars on soil greenhouse gas production. *Annals of environmental science* **3**, 179-193 (2009).

14. Tan, L. *et al.* Effect of three artificial aging techniques on physicochemical properties and Pb adsorption capacities of different biochars. *Science of The Total Environment* **699**, 134223 (2020).

15. Zhao, R., Coles, N. & Wu, J. Carbon mineralization following additions of fresh and aged biochar to an infertile soil. *CATENA* **125**, 183–189 (2015).

16. Báder, M., Németh, R., Sandak, J. & Sandak, A. FTIR analysis of chemical changes in wood induced by steaming and longitudinal compression. *Cellulose* **27**, 6811–6829 (2020).

17. Chen, H. *et al.* Qualitative and quantitative analysis of wood samples by Fourier transform infrared spectroscopy and multivariate analysis. *Carbohydrate Polymers* **82**, 772–778 (2010).

18. Delarue, F. *et al.* Can Rock-Eval pyrolysis assess the biogeochemical composition of organic matter during peatification? *Organic Geochemistry* **61**, 66–72 (2013).

19. Traoré, M., Kaal, J. & Martínez Cortizas, A. Differentiation between pine woods according to species and growing location using FTIR-ATR. *Wood Sci Technol* **52**, 487–504 (2018).
